# Polycatecholamine nanocoatings on stainless steel: the effect on attachment of human fibroblasts and platelets

**DOI:** 10.3762/bjnano.17.25

**Published:** 2026-02-20

**Authors:** Paulina Trzaskowska, Ewa Rybak, Maciej Trzaskowski, Kamil Kopeć, Jakub Krzemiński, Rafał Podgórski, Hatice Genc, Mehtap Civelek, Iwona Cicha

**Affiliations:** 1 Centre for Advanced Materials and Technologies CEZAMAT, Warsaw University of Technology, Poleczki 19, 02-822 Warsaw, Poland; 2 Faculty of Chemical and Process Engineering, Warsaw University of Technology, Warynskiego 1, 00-645 Warsaw, Poland; 3 Section of Experimental Oncology and Nanomedicine, Department of Otorhinolaryngology, Head and Neck Surgery, Universitätsklinikum Erlangen, Glueckstraße 10a, 91054 Erlangen, Germany

**Keywords:** cell–material interactions, Fenton oxidation, hemocompatibility, nanocoatings, polycatechols

## Abstract

Polydopamine (PDA) is widely used to functionalize materials and enhance cell attachment. At the same time, the potential of the dopamine precursor tyrosine in its polymerized form (polytyrosine, PTYR) remains underexplored despite its biological activity. In this study, we developed nanostructured PDA and PTYR layers on stainless steel 316L via a novel in situ oxidation process and evaluated their physicochemical properties and cellular interactions at the nano/microscale. Surface characterization revealed that the polymeric coatings formed a homogenous layer with distinct topographical features and thickness in the nanometer range for PTYR and in the micrometer range in case of PDA. Compared to PDA, PTYR coatings exhibited a nanoparticulate surface morphology and higher stability under physiological conditions. Wettability, roughness, and amine group density were systematically analyzed to determine their influence on interactions with fibroblasts and platelets. Our results show that PTYR nanocoatings significantly reduced platelet adhesion and activation, whereas PDA coatings, due to their higher primary amine content, could in some cases enhance platelet adhesion. Furthermore, fibroblast attachment was primarily influenced by coating roughness, with a specific threshold beyond which adhesion did not increase or was negatively impacted. These findings highlight the potential of engineered PTYR nanocoatings for developing advanced hemocompatible surfaces for biomedical implants.

## Introduction

Stainless steel 316L (SS 316L) is an iron-based alloy containing chromium and molybdenum, which promote passivation and corrosion resistance [1]. Owing to its mechanical robustness, manufacturability and relatively low cost, SS 316L is widely used for orthopedic and dental implants, bone plates, screws, oral implants, and vascular stents [1–5]. In dental and orthopedic applications, fibroblast attachment, spreading, migration, and osteointegration are critical for long-term implant stability. Consequently, a variety of surface modifications have been developed to improve cell–material interactions, including anodized nanopit arrays, hydroxyapatite–collagen layers on polydopamine-modified steel, nanoporous coatings influencing integrin/ERK signaling, and bilayers comprising graphene oxide and forsterite nanoparticles, all of which were shown to enhance fibroblast adhesion, migration, or proliferation [2–5].

Beyond orthopedic and dental uses, SS 316L is a standard material for cardiovascular implants, particularly stents, due to its strength, ductility and ability to form thin, expandable struts. However, bare SS 316L stents exhibit limited hemocompatibility and are susceptible to in-stent restenosis and thrombosis [6–7]. Early and complete endothelialization plays a crucial protective role in preventing these complications, as endothelial cells restore vascular homeostasis and suppress thrombogenic and proliferative responses [6]. Under long-term hemodynamic loading, stainless steel implants may also suffer from localized corrosion and metal-ion release, leading to chronic inflammation at the tissue–implant interface [8].

While rapid colonization of implant surfaces with native cells promotes their integration and mitigates inflammatory processes, the coatings on long-term metallic implants must fulfil some essential requirements. Such coatings must (i) provide corrosion protection, (ii) maintain mechanical integrity, (iii) support cell–material interactions, and (iv) reduce bacterial adhesion and thromboinflammatory responses [9–11]. In terms of stability, coating damage, such as cracking, delamination or peeling, not only impairs healing, but may also enhance platelet attachment, or release fragments into the bloodstream [12]. Thus, there is a clear need for durable, biocompatible, and hemocompatible coatings to improve the long-term safety of stainless steel implants.

Polydopamine (PDA) has emerged as a versatile coating material for cardiovascular devices, tissue engineered scaffolds and antimicrobial medical devices [13–17]. PDA coatings obtained by self-polymerization at mildly basic pH, or by oxidative routes, improve cell adhesion and proliferation [18], reduce bacterial adhesion by more than 90% [16,19], and markedly enhance hemocompatibility [20]. Long-term stability has been demonstrated through in vivo persistence over several weeks and prolonged antimicrobial activity [21–23]. Beyond classical PDA films, broader catechol-based and PDA-inspired chemistries have been explored, including phenolic polymer systems, mussel-inspired adhesive interfaces and catechol-rich surface architectures, which have been shown to modulate protein adsorption and cellular responses at the biointerface [24–25]. Despite this broad interest in catechol-based coatings, alternative polycatecholamines remain only minimally explored. To date, only two studies have specifically examined polytyrosine (PTYR), or structurally related polycatecholamines, as standalone coating systems [12,26]. Their results indicated that polycatecholamines may provide tunable surface properties distinct from those of conventional PDA films.

**Figure 1:**
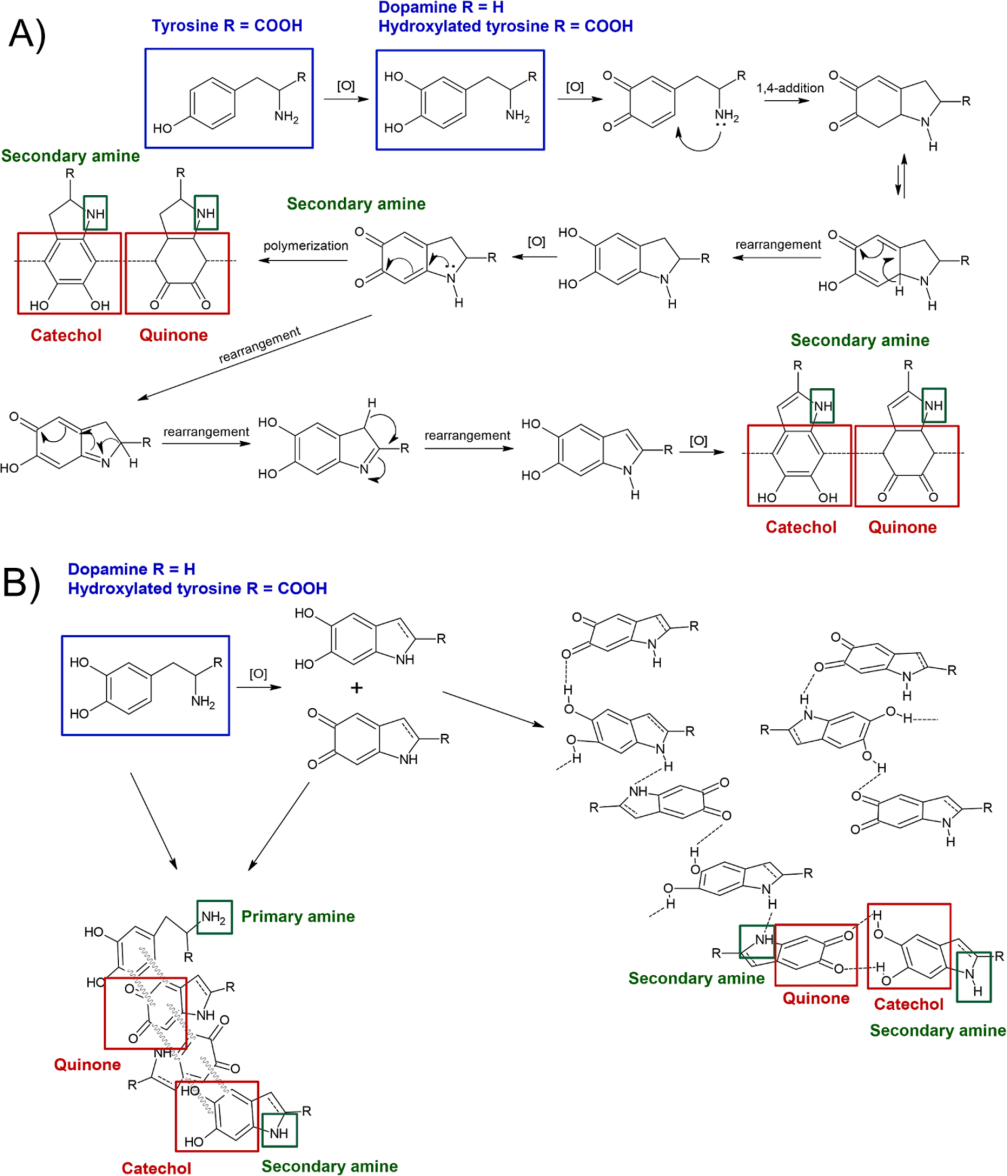
Schematic presentation of polycatechol polymerization. (a) Anticipated mechanism of ʟ-tyrosine and ʟ-dopamine polymerization and (b) proposed non-covalent interactions between monomers in oxidative polymerization of ʟ-dopamine and hydroxylated ʟ-tyrosine. Figure 1 was redrawn from [12] with the use of ChemSketch (ACD/Labs).

Although PDA and PTYR originate from different amino acid precursors, both polymers are believed to undergo oxidation-driven formation of catechol–quinone intermediates, followed by intramolecular cyclization and supramolecular aggregation [12,27]. Figure 1 summarizes the hypothetical steps of dopamine and tyrosine oxidation, illustrating how both monomers may converge toward heterocyclic structures and supramolecular aggregates stabilized by charge transfer, hydrogen bonding and π–π stacking interactions [12,27–28]. Mechanistic studies indicate that the first step of ʟ-tyrosine polymerization involves hydroxylation of the aromatic ring at the C3 position [12,28–29], after which the pathway resembles dopamine oxidation. The resulting polymers contain mixtures of catechol and quinone moieties, as well as primary and secondary amine groups, whose relative proportions depend on the polymerization conditions [12,27–29]. The differences in monomer structure and oxidation route (e.g., Fenton-type vs autoxidation) are expected to influence the catechol/quinone balance, amine content, nanostructure and, consequently, the biological and hemocompatible properties of the resulting coatings.

In this study, we applied a novel in situ oxidation process to obtain nanostructured PDA and PTYR coatings on SS 316L, using a Fenton-type reaction for PTYR synthesis and previously optimized conditions for PDA deposition [12,27]. We compared the physicochemical properties of these coatings and evaluated their effects on L929 fibroblasts, human dermal fibroblasts, and human platelets. By directly comparing PDA with the much less explored PTYR under identical conditions, we aim to elucidate how the differences in polycatecholamine chemistry and nanostructure translate into cell–surface interactions on stainless steel implants.

## Results

### Wettability of the coatings

**Figure 2:**
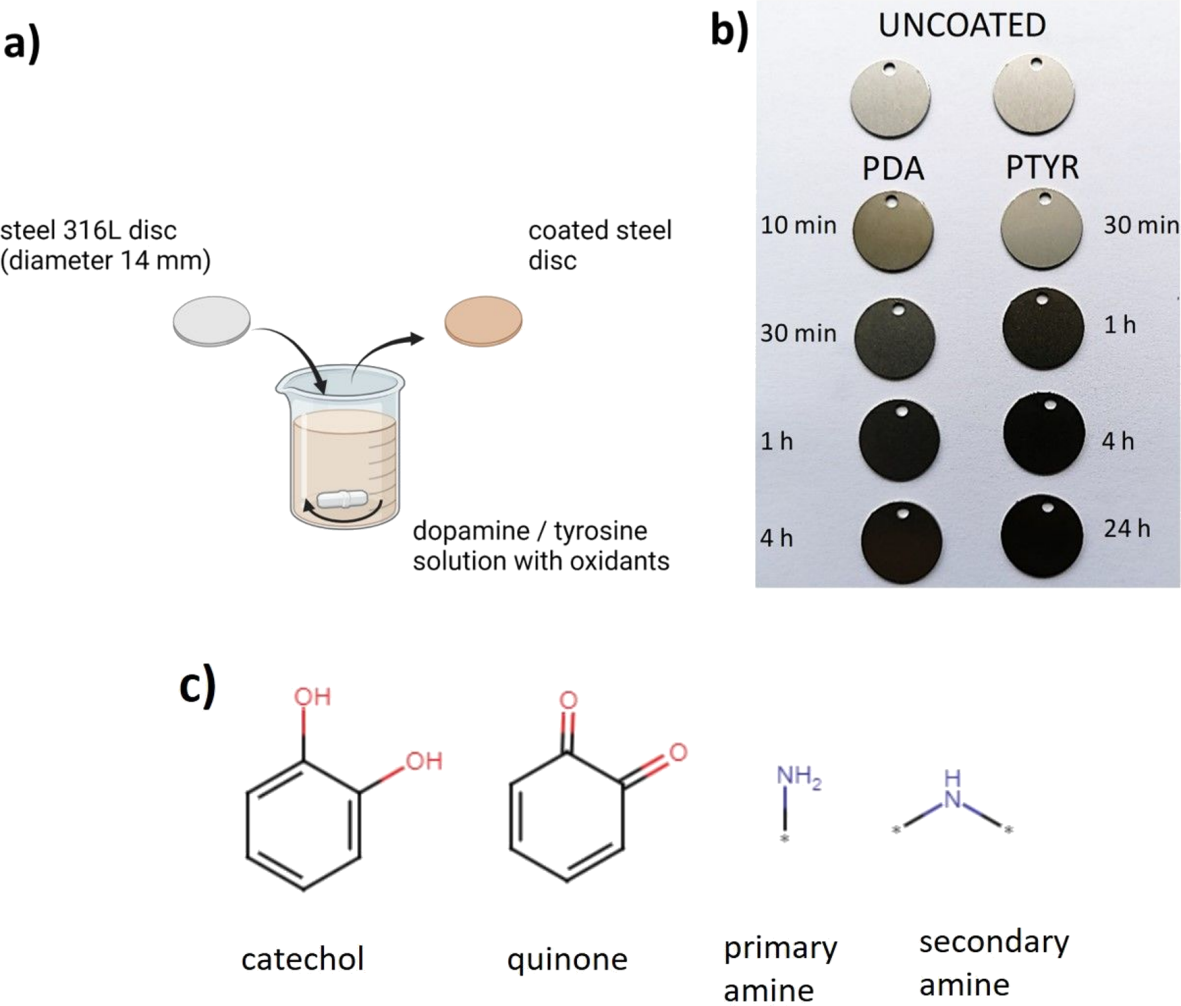
Coating process and appearance of samples. (a) Schematic presentation of the coating process setup, (b) appearance of coatings variants, and (c) possible chemical groups present in polycatecholamines. Figure 2a was created in BioRender. Trzaskowska, P. (2026) https://BioRender.com/o2f9etk This content is not subject to CC BY 4.0.

Polished SS 316L discs were coated with PDA (reaction times: 10 min, 30 min, 1 h, or 4 h) or PTYR (reaction times: 30 min, 1 h, 4 h, or 24 h), according to the schematic presentation of the coating process shown in Figure 2a. The resulting appearance of the coating variants is shown in Figure 2b. The longer the time of reaction, the darker were PDA and PTYR coatings. Important chemical groups in polycatecholamine coatings (i.e., catechol, quinone, primary amine, and secondary amine groups) relevant to the reaction scheme from Figure 1 are shown in Figure 2c. Both the images of the obtained samples (Figure 2b) and their CA values (Table 1) indicate that, regardless of the coating time, the coatings were successfully introduced on the SS 316L surface. Photos of the samples shown in Figure 2b show that both PDA and PTYR coatings get darker with increasing coating time, reflecting the growing thickness of coatings. As anticipated from the literature data presented in the Introduction section, PDA and PTYR coatings consist of catechol and quinone groups, as well as primary and secondary amine groups (Figure 2c).

**Table 1:** Contact angle (CA) values measured for SS and each type of coating. SD – standard deviation.

SS		SS-PDA			SS-PTYR		
		
CA [°]	SD	coating time	CA [°]	SD	coating time	CA [°]	SD

99.40	3.61	10 min	52.97	7.19	30 min	42.32	10.80
		30 min	40.10	8.09	1 h	40.93	10.23
		1 h	21.22	6.06	4 h	27.83	3.40
		4 h	11.89	2.86	24 h	23.00	6.98

Contact angle (CA) values presented in Table 1 indicate that the SS 316L surface without any modifications is hydrophobic (99.40°), whereas all PDA and PTYR coating variants led to a significant decrease in CA. The decrease in CA is caused by many hydrophilic chemical groups incorporated in PDA and PTYR coatings, for example, hydroxy or amino groups. In the case of all materials, the longer the coating time was, the more hydrophilic the surface became. For SS-PDA, the shortest time of coating resulted in a CA of 52.97°. The longest coating duration decreased the CA to 11.89°. Similarly, PTYR coating led to hydrophilic surfaces, but the CA decreased only to 23.00° after the coating process of 24 h. The materials obtained with extreme coating times were subjected for further tests, that is, PDA 10 min, PDA 4 h, PTYR 30 min, and PTYR 24 h.

### Thickness of the coatings

**Figure 3:**
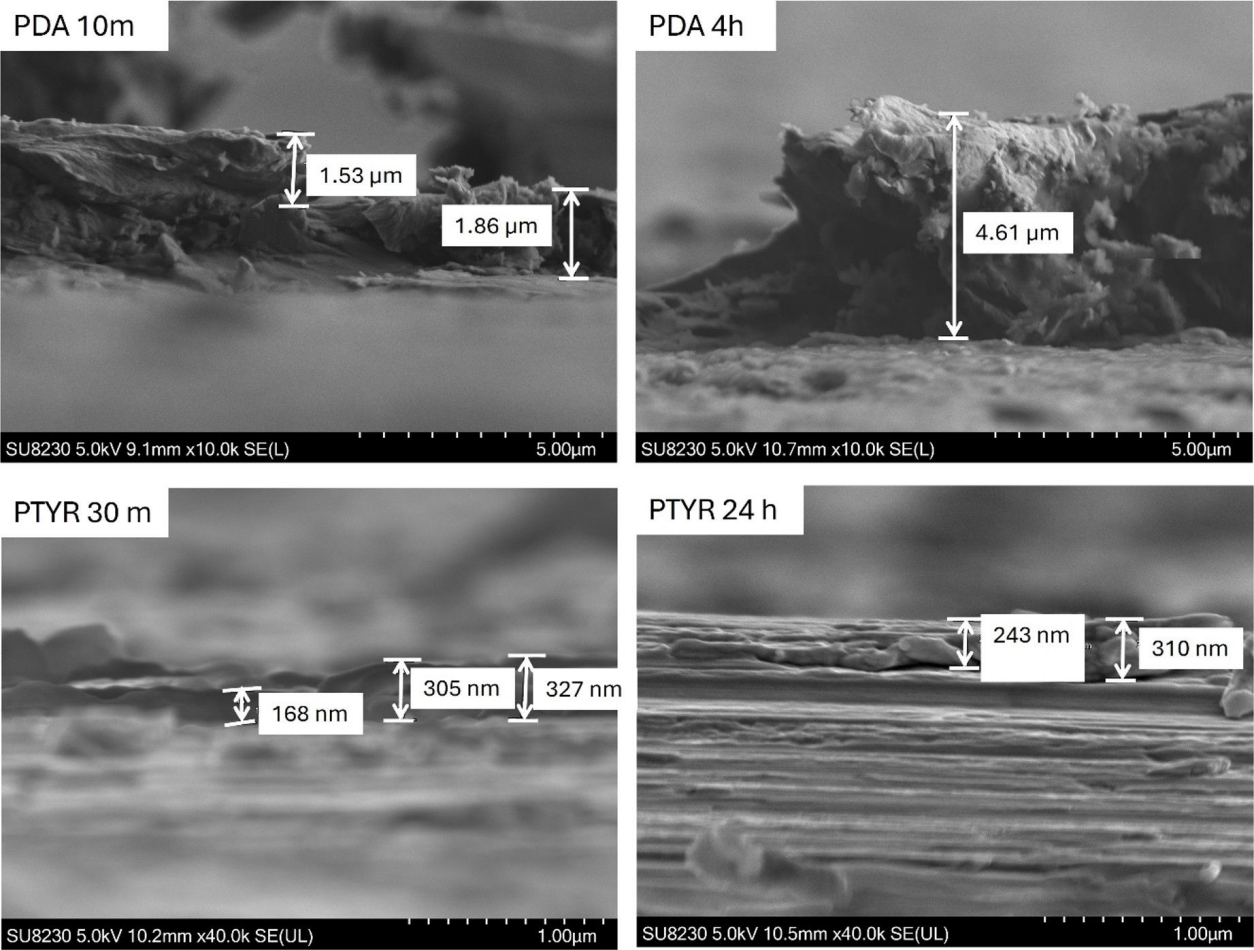
SEM images of coating variants in the scratch location, together with example thickness measurements.

Thickness of the coatings was measured with SEM thanks to the tilt of the samples in the scratch location (Figure 3). The average thicknesses of PDA 10 min and 4 h coatings were 1.58 and 4.09 µm, respectively. For PTYR, much thinner coating layers were measured, with a mean value of 308.78 nm (range: 168–441 nm) for SS-PTYR 30 min and mean value of 360.86 nm (range: 243–637 nm) for SS-PTYR 24 h. As expected, the thickness of PDA and PTYR layers increased with the coating time, but the increase was very slow in case of PTYR coating, whereas the thickness of PDA layer increased more than 2.5 fold within only 4 h.

### Roughness analysis

**Figure 4:**
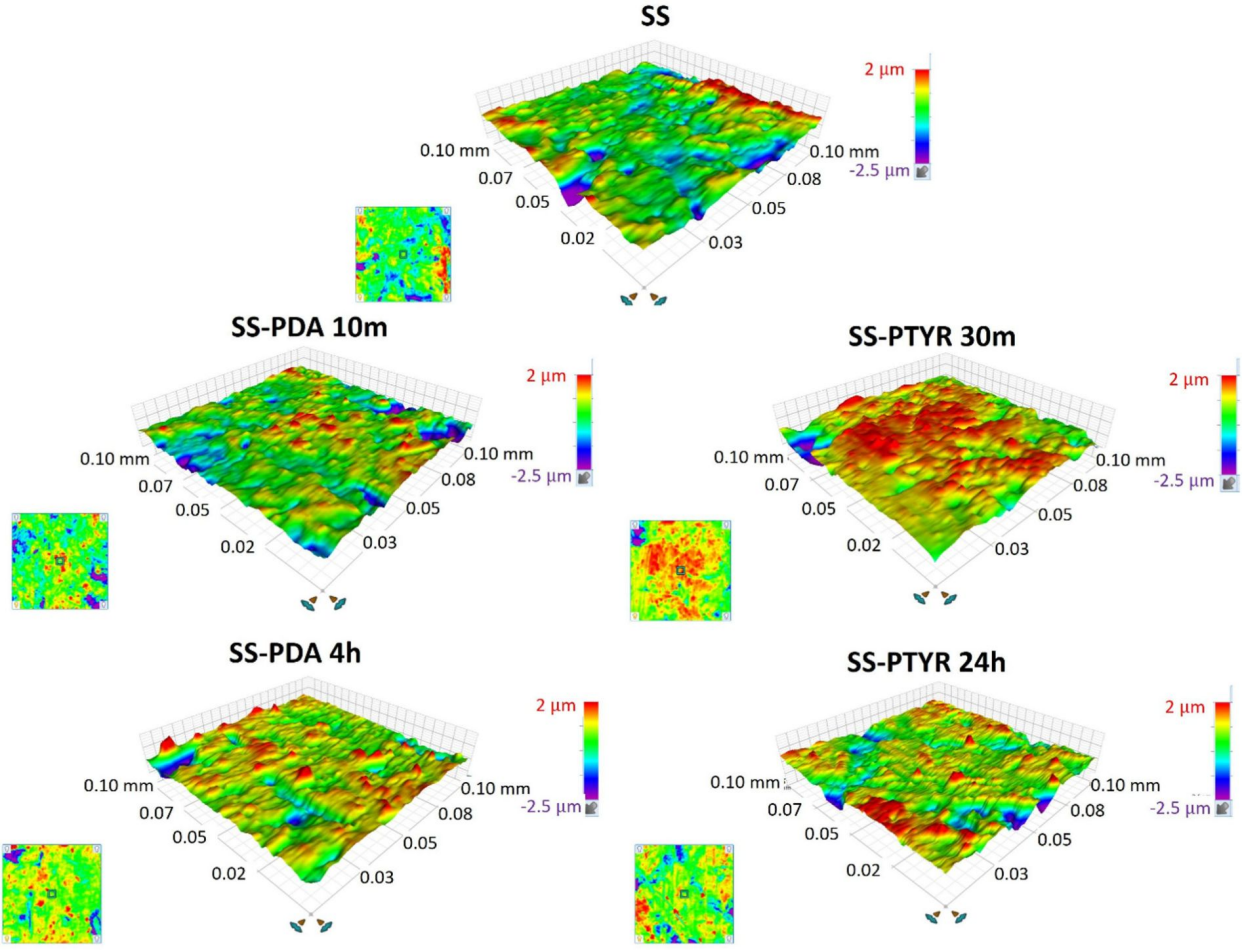
3D graphs of surface profiles generated by the profilometer software showing the peaks and depths on the coated surfaces compared to uncoated SS 316L.

In comparison to the uncoated SS 316L surface, Ra values reflecting roughness average were barely altered after coating (Table 2). Among the analyzed samples, only the SS-PTYR 24 h variant was characterized with a noticeably higher Ra (0.31 µm) than the control, which can be related to polytyrosine nanoparticles irregularly distributed on the surface. Rku values, representing the sharpness of the profile, also changed only slightly after coating for all variants. The exception is SS-PDA 4 h, for which the Rku value increased to 6.14 µm (Table 2). The higher the Rku value, the deeper and vaster depths are present on the surface; thus, for this variant, the surface area is more developed, that is, the total surface area is larger because of many nanoscale peaks and depths. The 3D graphs of surface profiles generated by the profilometer software are provided in Figure 4.

**Table 2:** Roughness parameters (Ra and Rku) for each coating variant.

Coating variant	Ra [µm] “roughness average”	Rku [µm] “profile sharpness”

SS	0.20 ± 0.07	3.91 ± 1.54
SS-PDA 10 min	0.18 ± 0.04	3.55 ± 2.05
SS-PDA 4 h	0.24 ± 0.13	6.14 ± 5.04
SS-PTYR 30 min	0.20 ± 0.06	4.41 ± 1.52
SS-PTYR 24 h	0.31 ± 0.15	3.51 ± 1.40

### Sterilization effectiveness of the materials

Both tested sterilization methods for steel discs with PDA and PTYR coatings were effective. The exact method description and more detailed results are provided in Supporting Information File 1.

#### Coating resistance under physiological conditions

**Figure 5:**
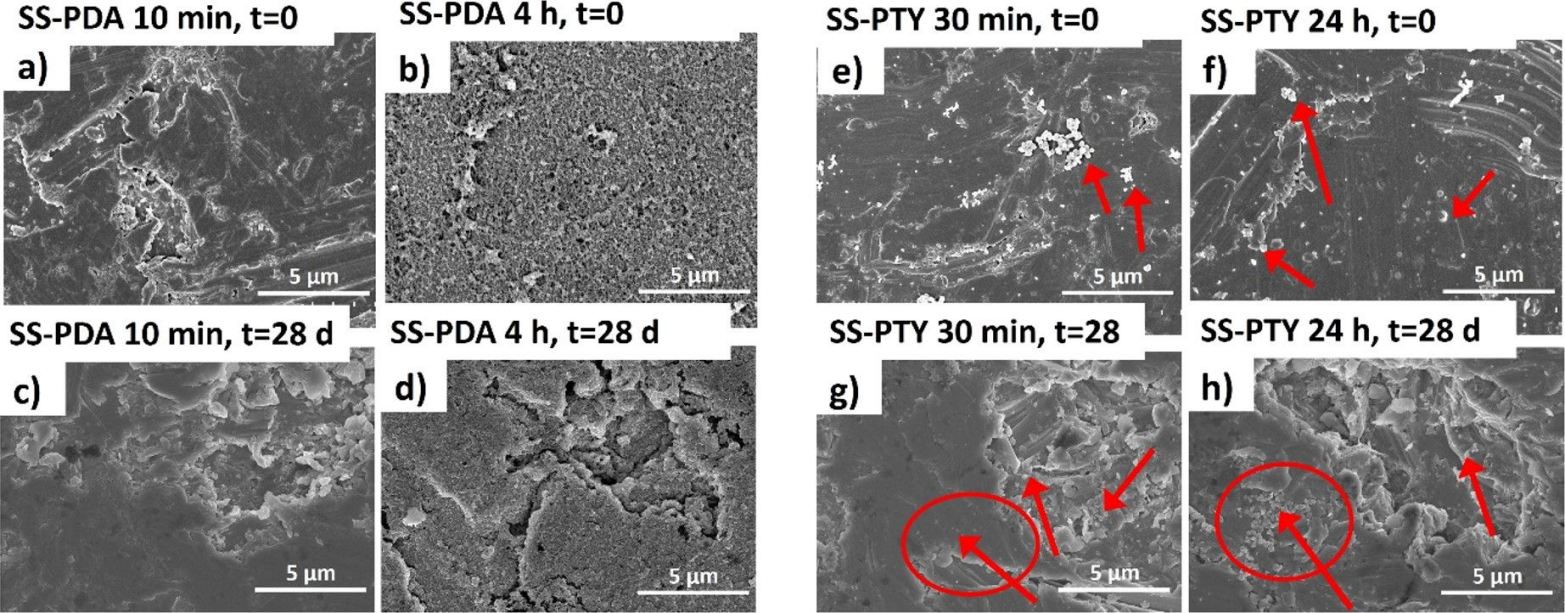
SEM images (magnification 5 000×) of selected coatings surfaces ((a) SS-PDA 10 min, SS-PDA 4 h and (b) SS-PTYR 30 min, and SS-PTYR 24 h) just after coating (*t* = 0) and after 28 d of incubation in PBS. Arrows indicate PTYR nanoparticles present on SS-PTYR surfaces.

While all coatings variants underwent the washing resistance test, only images of selected ones are presented in Figure 5, namely, the PDA and PTYR coatings with the shortest and longest time of coating. Images taken directly after coating (Figure 5a,b,e,f) show the initial coating morphology. The surface of SS-PDA 10 min could not be clearly distinguished with SEM; however, the surface of SS-PDA 4 h presented a full coverage with a polymer film. After 28 d of constant washing with PBS, the coating is still present on the SS-PDA 4 h variant, although it seems to be cracked. The coating presence on the SS-PDA 10 min variant could not be confirmed with SEM after 28 d of washing. The morphology of PTYR nanocoatings was significantly different. In Figure 5e,f, bright micro/nanoparticles are visible, presumably consisting of PTYR (indicated by the red arrows). In contrast, the SS-PTYR 24 h coating seemed more homogenous. After the 28-day washing period, the particles were still present; thus, it was assumed that the PTYR coating both on SS-PTYR 30 min and SS-PTYR 24 h endured the washing test.

#### Primary amine content evaluation

**Figure 6:**
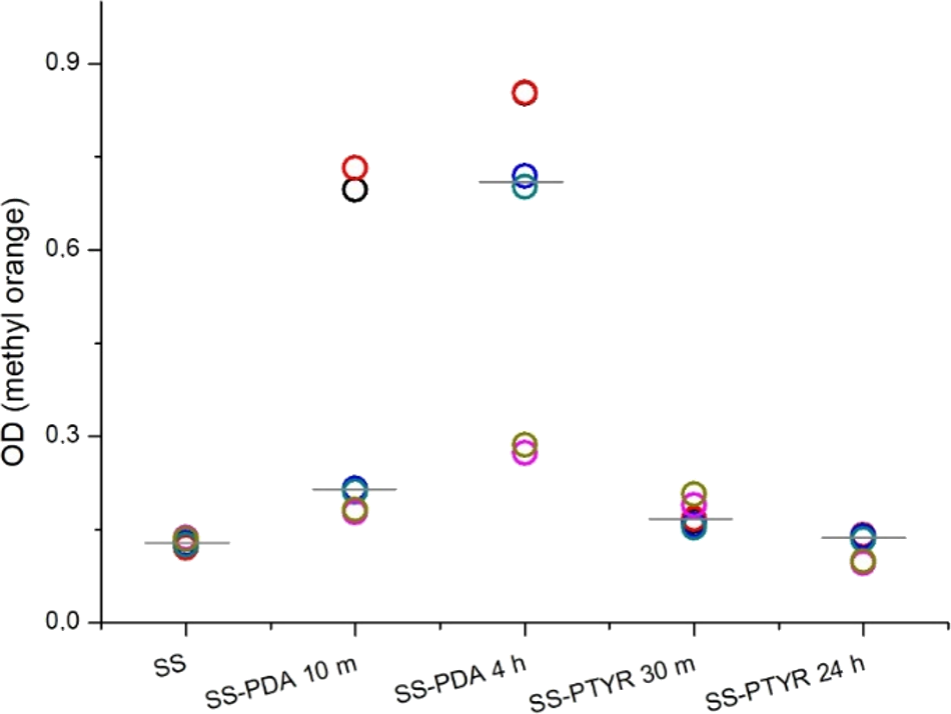
Amount of amine groups in the tested coatings. The mean OD values taken from each repetition of the analysis are presented as open round symbols on a scatter plot. Thin grey lines represent the median OD values observed for each material variant.

Figure 6 shows the primary amine group content in the analyzed coating variants. The level of amine groups varied strongly in PDA 10 min and PDA 4 h variants, whereas in other samples it was more reproducible. Also, PDA coatings were characterized with the biggest –NH_2_ amount. It must be noted that the coating thickness does not determine the –NH_2_ content available for reaction with methyl orange since the reaction happens on the material surface.

#### Cytotoxicity towards L929 cells

**Figure 7:**
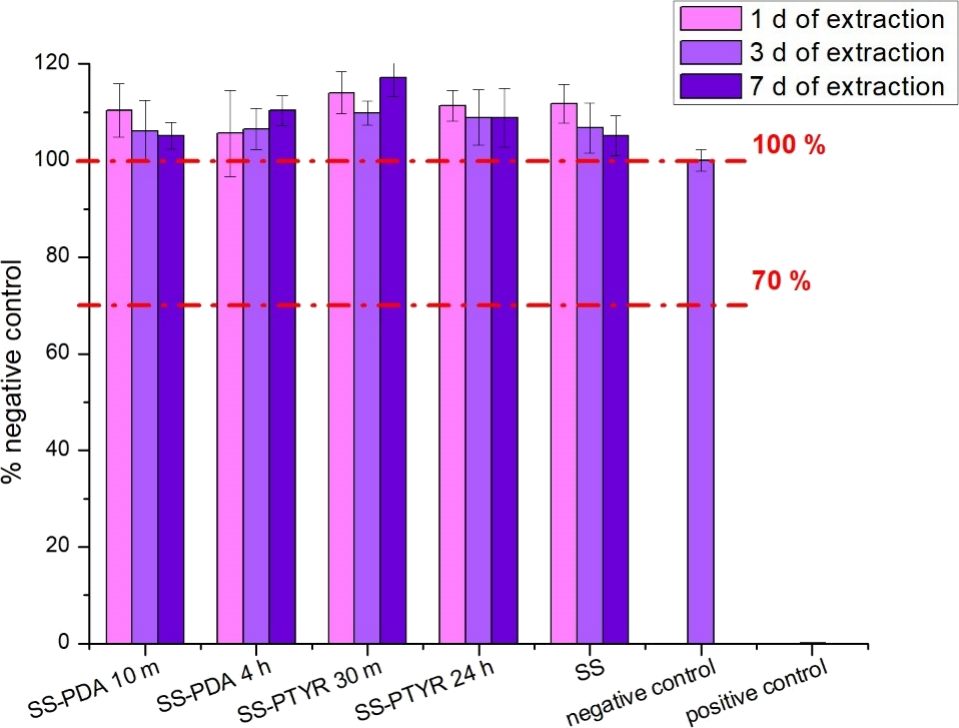
Cytotoxicity of the coatings evaluated with MTT test on materials extracts expressed as mean % of the negative control. Three extraction times were applied: 1, 3, and 7 d.

According to ISO 10993-5, materials are non-cytotoxic if their cytotoxicity values obtained with MTT test exceed 70%. Our results (Figure 7) show that in our case, all extracts regardless of extraction time were nontoxic, meaning that none of the tested coating extracts had a negative effect on cell viability.

#### Human dermal fibroblasts adhesion

**Figure 8:**
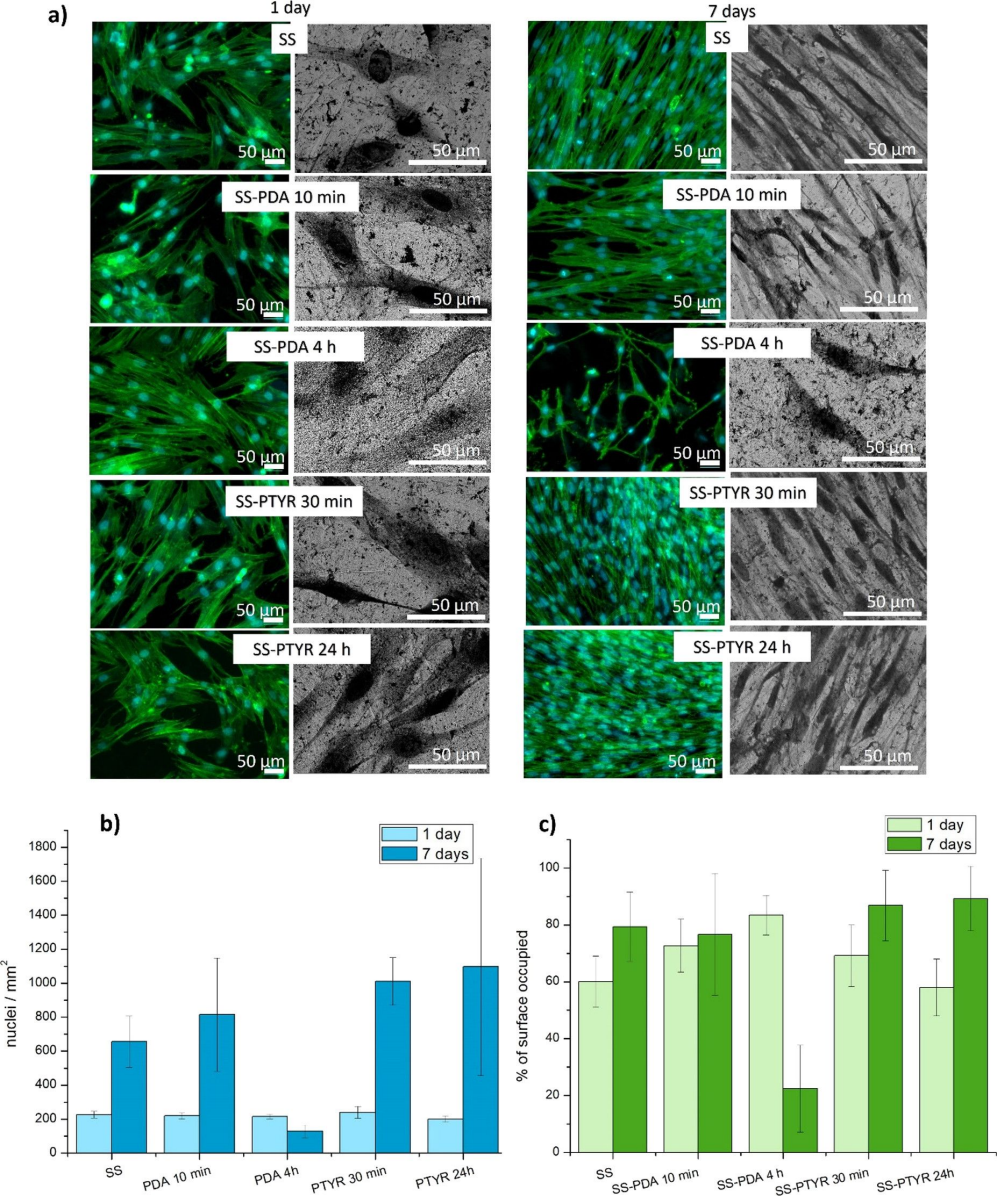
Adhesion of human fibroblasts to materials’ surfaces. (a) Confocal and SEM images of fibroblast adhered to the materials surface after 1 or 7 d of culture: green, F-actin; blue, nuclei. For each sample, six independent fields of view (FOVs) were analyzed. A single FOV corresponded to an area of 184,500 µm^2^, yielding a total analyzed area of approximately 1.11 mm^2^. (b) Mean percentage of nuclei per square millimeter on each coating variant after 1 or 7 d of culture. (c) Mean percentage of surface occupied by fibroblasts on each coating variant after 1 day or 7 d of culture.

Microscopic images in Figure 8 indicate typical healthy fibroblast morphology, for example, spindle-shaped cells. After 7 d, the cells formed thick layers covering the most of materials surface, with the largest percentage of cell-covered surfaces for nanocoated SS-PTYR 30 min (86.82%) and SS-PTYR 24 h (89.25%). As shown in Figure 8b,c, the total cell number and surface occupied by cells as a result of migration and proliferation increased with culture time for each coating variant, apart from SS-PDA 4 h, on the surface of which these values decreased significantly (from 83.42% to 22.45%). SS-PDA 4 h was also the variant characterized by the lowest CA (11.89°) and highest Rku (6.14 µm) among all samples. SEM images shown in Figure 8a indicate that there was no cellular preference to adhere to any specific morphologic spots. Table 3 presents correlations between cell number and cell total surface and different physicochemical parameters. Additional fluorescent images of fibroblasts adhered on the surfaces, stained with N-cadherin are presented in Supporting Information File 1, Figure S3.

**Table 3:** Correlation between the adhered fibroblast parameters (after 1 and 7 d of culture) and surface parameters (Ra, Rku, and CA) expressed as Pearson’s correlation coefficient (*r*).

	*r*-Value^a^			
	
parameter	% surface covered by fibroblasts (day 1) [mm^2^]	% surface covered by fibroblasts (day 7) [mm^2^]	fibroblast nuclei number (day 1) [mm^2^]	fibroblast nuclei number (day 7) [mm^2^]

Rku “sharpness of the profile”	0.80	−0.91	0.12	−0.84
Ra “roughness average”	−0.31	−0.28	−0.81	0.13
CA	−0.50	0.44	0.44	0.15

^a^*r*-Values from 0 to 0.25 or from 0 to −0.25: no correlation, from 0.25 to 0.50 or from −0.25 to −0.50: poor correlation, from 0.50 to 0.75 or −0.50 to −0.75: moderate to good correlation, from 0.75 to 1 or from −0.75 to −1: very good to excellent correlation.

#### Adhesion and activation of human platelets

**Figure 9:**
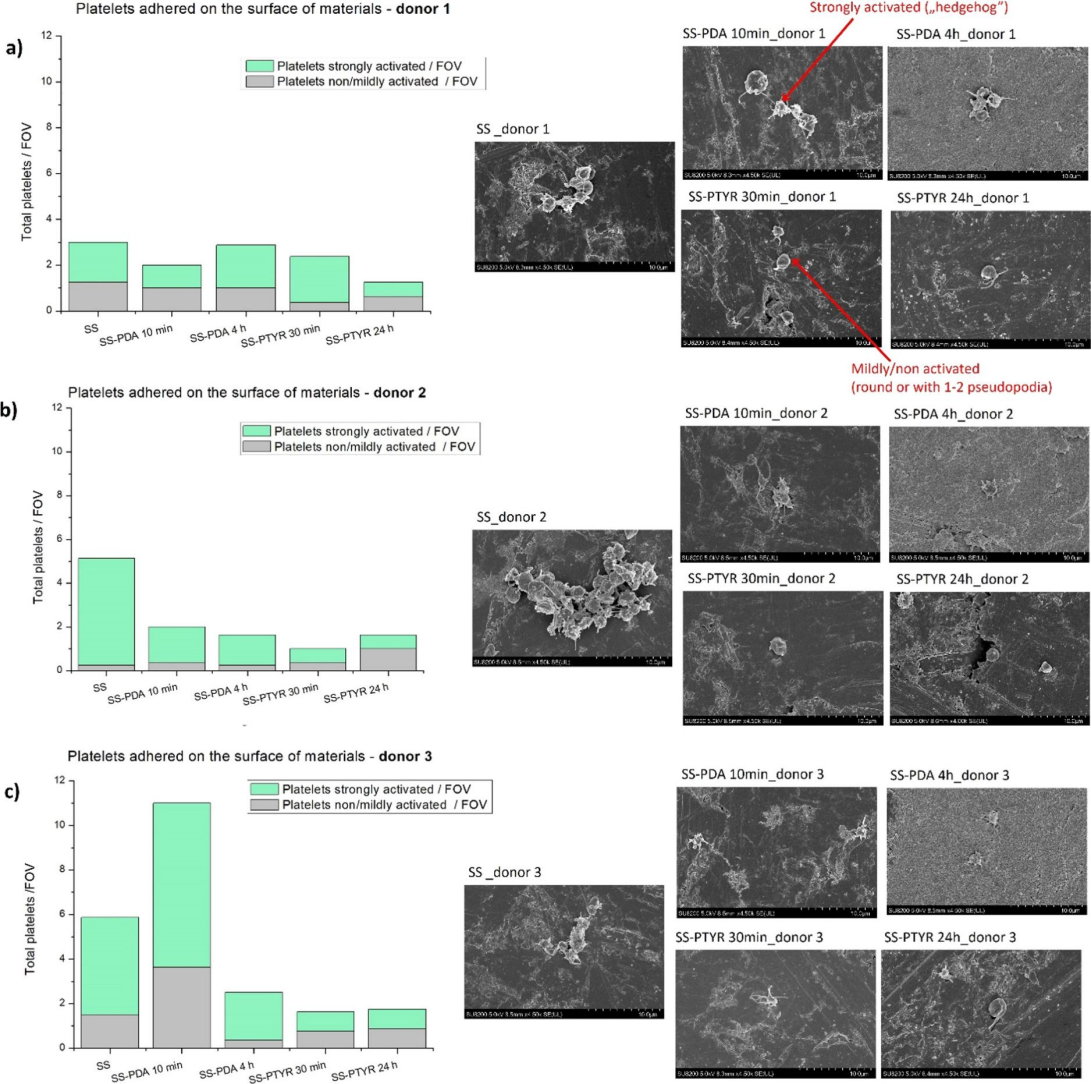
Platelet adhesion to the coated materials. Left: Platelets obtained from donors (a) 1, (b) 2, and (c) 3 were counted per FOV according to the activation level. For each sample, eight FOVs were acquired. A single FOV corresponded to an area of 3162 µm^2^, resulting in a total analyzed surface area of 25 296 µm^2^ per sample. Right: SEM images at a magnification of 500×. Exemplary appearances of a strongly activated and a mildly activated platelet are indicated on the SEM images obtained from the donor 1 sample.

Platelet adhesion to the materials was visualized with SEM, and cells were counted while distinguishing mildly and strongly activated ones. Because platelet reactivity varies individually, platelet-rich plasma (PRP) obtained from three donors was independently tested. Figure 9 presents the evaluation of the material coating effect on platelet attachment and activation. In the case of donor 1, there were few platelets present on the tested materials, and the smallest amount was observed on nanocoated SS-PTYR 24 h. Platelets from donor 2 were attracted to the SS 316L surface stronger than to all SS-PDA and SS-PTYR variants, whereby the lowest number of strongly activated platelets was observed on SS-PTYR. For donor 3, the highest amount of both adhered and activated platelets was observed, especially on the SS-PDA 10 min variant. Adhesion and activation of platelets were reduced on other variants (see also Supporting Information File 1, Figure S2). For every donor, the PTYR 24 h nanocoating prevented platelet activation the most, since the number of strongly activated platelets was persistently the lowest for this coating variant.

## Discussion

This study aimed to establish the conditions for the potential application of nanostructured PDA and PTYR coatings on SS 316L with two different functionalities, namely, as a cell adhesion-promoting surface for better implant integration with surrounding tissues and as a platelet adhesion-repelling surface to reduce the risk of thrombosis upon contact with blood.

Compared with extensively investigated PDA, little has been known until now about PTYR’s potential to serve as a biomedical coating. In a previous study from our groups, coatings derived from ʟ-tyrosine, ʟ-phenylalanine, and 2-phenylethylamine were shown to substantially reduce water contact angles by 50–80%, indicating a pronounced increase in surface hydrophilicity [12]. Thus, we now focused on the comparison of PTYR and PDA coating properties, demonstrating that, although chemical composition of both might be assumed to be very similar, we were able to distinguish significant differences.

For PDA and PTYR coatings, we applied a chemical oxidation process much faster than the typically used atmospheric oxidation (because the amount of oxidant in the solution is not limited by its dissolution) and allows for precise control over coating uniformity and thickness at the nanoscale. Also, for the synthesis of PTYR coatings, there is a limitation in the substrate availability for the oxidation reaction due to the limited solubility of ʟ-tyrosine in water. Thus, we used a supersaturated ʟ-tyrosine solution, to ensure that the oxidized part of the substrate is replaced by a fresh portion dissolved from the suspension [12]. The resulting SS-PDA and SS-PTYR samples were sterilizable, non-toxic, and highly hydrophilic. The observed decrease in CA with the coating time could be explained by the progressive filling of micro- and nanoscale surface irregularities, leading to increased homogeneity.

SEM was the only feasible technique enabling the measurement of the thickness of these coatings. Such measurements are rarely reported. PDA coatings produced via oxidation on metallic biomaterials measured between 2 nm and nearly 1 µm. Reports include Cu^2+^ oxidation, yielding coatings greater than 70 nm and simple O_2_ oxidation giving 45 ± 5 nm [17], KMnO_4_ treatment resulting in approximately 33 nm [30], and autoxidation in basic solution leading to films 2 nm or less [31–32].

In contrast, immersion processes produce approximately 1 µm films [33]. These results differ from our PDA coating thickness values due to divergent reaction parameters and, most probably, distinct initial surface roughness. So far, there are no studies reporting explicit thickness measurements for PTYR coatings, which were in nanometer range even after 24 h coating process in our study.

The in vitro experiments indicated that human dermal fibroblasts could migrate and spread on both types of surfaces. But after 7 d on culture, one variant (SS-PDA 4 h) did not provide sufficient base for cell anchorage. SS-PDA 4 h was characterized by the lowest CA and the highest Rku. This suggests that an excessively hydrophilic surface combined with pronounced nanoscale roughness prevented cells from proper adhesion via integrins and, at the same time, high Rku with sharp nanofeatures hindered cell migration. As a result, there was no sufficient cell–cell interaction, which may lead to the detachment of cells [34]. It was reported that surfaces characterized by low CA values do not support fibroblast adhesion. Kim et al. reported that 50–60° is the optimal CA value for adhesion and proliferation of fibroblasts on low-density polyethylene surfaces [35]. According to Webb et al., however, moderately hydrophilic surfaces (20–40°) promoted the highest levels of fibroblast attachment [29]. In our study, CAs ranging from 52.97° to 23.00° promoted fibroblast spreading and growth during 7 d of culture (SS, SS-PDA 10 min, SS-PTYR 30 min, SS-PTYR 24 h). In contrast, the CA of 11.89° on SS-PDA 4 h was too hydrophilic, and cells detached after 7 d.

**Figure 10:**
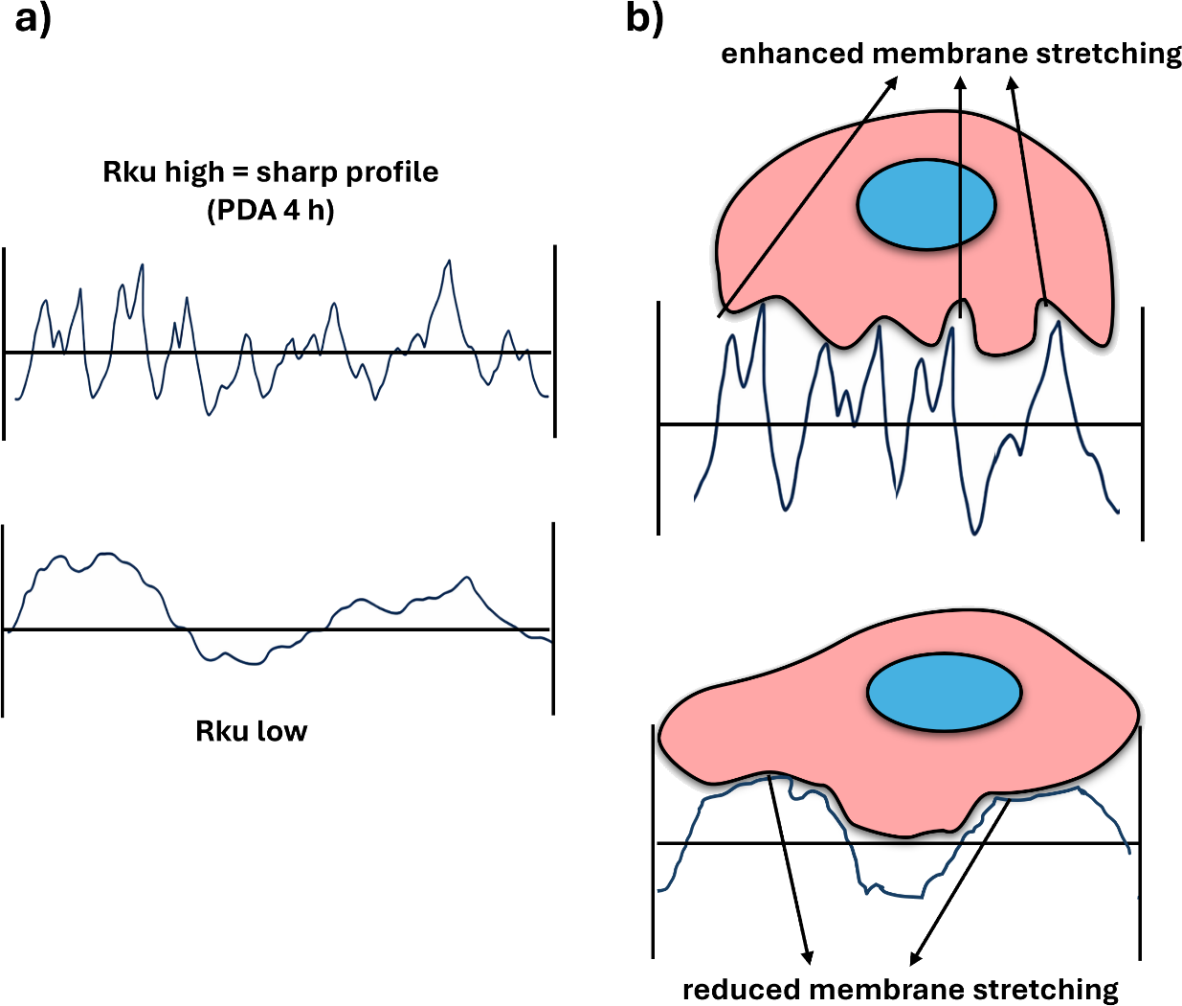
Effect of Rku on cell attachment. (a) Graphical explanation of high-Rku and low-Rku profiles. (b) Cellular membrane stretching on high-Rku (top) and low-Rku (bottom) surfaces. Figure 10a was redrawn from [45]. Figure 10b was redrawn from [28].

Rku is rarely taken into consideration in studies concerning the surface effect on cell attachment. However, in our study, Rku was the most dominant factor affecting fibroblast spreading and attachment. Rku describes the character of nanoscale peaks and valleys on the surface (see Figure 5 and Figure 10a). The higher Rku, the sharper the profile is [36].

We found that Rku correlated very well with the surface area covered by fibroblasts. The dependence was directly proportional at the beginning of culture, and inversely proportional after 7 d. Figure 10b shows the schematic presentation of the different effects of high-Rku and low-Rku profiles on cell membrane stretching. In line with this, Pearson’s correlation coefficient values shown in Table 3 indicated a strong positive correlation between Rku and surface coverage by fibroblasts after 1 d of culture (0.80) and an even stronger negative correlation between Rku and surface occupied by fibroblasts after 7 d (−0.91). We also determined a strong negative correlation between Rku and cell number after 7 d of culture (−0.84). These two correlations indicate that the sharper the surface profile is (more sharp depths and peaks), the fewer cells are attached after 7 d of culture, while the initial adhesion was not affected by the higher Rku.

Further, a strong negative correlation between Ra and cell number after 1 d (−0.81) was determined, but it did not translate into any dependence after 7 d (0.13). Ra represents average roughness, which means the deviation of a surface from a mean height. A threshold-like behavior is also observed in the dependencies of proliferation and roughness as it has been established that high roughness deteriorates cell division rate [37]. But here only the initial proliferation could be strongly linked to Ra. After 7 d of culture, there was no correlation between Ra and cell number; however, cells multiplied, as shown in Figure 8b. Similar to our study, surface Ra affecting the initial threshold has been reported by other authors [38].

It is known that both nano/microroughness and wettability of the material surface can influence cell adhesion since these parameters affect the adsorption kinetics of the medium proteins to the surfaces and cells interact with surfaces through the protein layer [39]. Many previous reports demonstrated that grooves on the surface enhance fibroblast adhesion [28,40–41], but it has also been established that there is a roughness threshold for cell attachment that depends both on the cell type and other surface properties. Human dermal fibroblasts are characterized by large size and relatively low stiffness; thus, at the initial adhesion stage, they favor larger attachment areas (on the peaks of the surface). However, due to the large size and low stiffness, their cell membrane is strongly stretched, which deteriorates subsequent spreading and firm attachment [28] (Figure 10b). We speculate that this effect was responsible for the detachment of fibroblasts observed on the SS-PDA 4 h coating variant in our study (see Supporting Information File 1, Figure S3). The roughness threshold has previously been observed for fibroblasts also on silicone surfaces [42] and on various metallic surfaces [43], as well as for bone marrow stem cells on polycarbonate membranes [44].

Platelet adhesion and activation on artificial surfaces are triggered by the initial adsorption of pro-thrombogenic plasma proteins, such as fibrinogen, which is present in PRP and was used for the study [46]. This can lead to the formation of fibrin clots on the biomaterial surface [47]. To date, PDA coatings prepared using a traditional way (slow air oxidation) have been known for increasing the adhesion of platelets [48–49]. This was explained by PDA’s potential to bind any type of molecule, including fibrinogen, due to the chemical structure of PDA containing aromatic rings and reactive chemical groups. However, in the literature, there are no reports on PTYR nanocoatings regarding platelet adhesion. Our results demonstrated that, using plasma from different donors, many adhered and activated platelets were detectable on bare SS 316L. This was most probably caused by the hydrophobicity of this surface as SS with an untreated surface is known to attract platelets [50]. SS-PTYR 24 h nanocoating reduced platelet activation in the plasma of each donor, but the numbers of attached platelets varied strongly on both SS-PDA variants. This cannot be explained only by the differences in roughness since Ra and Rku of SS-PDA 10 min did not stand out from the values of the other variants, unlike the SS-PDA 4 h variant. Because of this reason, we evaluated the influence of primary amine content on platelet behavior. We established that both SS-PDA coatings contained high amounts of –NH_2_ groups. A high –NH_2_ content could be linked to the dominance of supramolecular monomer aggregates forming in PDA (Figure 1b). Additionally, the high –NH_2_ content detected in PDA 4 h can be associated with a more developed surface area than in other variants. The differences in platelet adhesion between SS-PDA 10 min and SS-PDA 4 h, both of which had high amine content, could therefore be related to the very low CA of the SS-PDA 4 h coating, which may have resulted in the inhibition of platelet attachment. The effect of primary amine content on platelet adhesion was also observed by other authors [22,51–52], who reported that high –NH_2_ content is not beneficial in terms of hemocompatibility because large amounts of primary amino groups (positively charged) are likely to promote platelet adhesion (negatively charged) by electrostatic interaction, as also observed in the case of SS-PDA 10 min variant. Moreover, low –NH_2_ content is linked to suppression of thrombogenicity since it enhanced maintaining the natural conformation of fibrinogen, which thereby inhibited platelet adhesion and activation [22,50,52], as observed in our study in the PTYR-coated samples.

## Conclusion

The results of our study indicated that PDA and PTYR coating layers on steel might be formed by slightly different mechanisms, which leads to distinct effects on platelet adhesion and activation. Thicker PDA coatings contained more primary amine groups, which was likely caused by the dominance of nanoscale supramolecular aggregates forming during the polymerization process. PTYR nanocoatings, containing fewer primary amine groups than PDA variants, exhibited nanoparticulate morphology, improved nanostructural stability, and were more repellent for platelets. This is an important finding in terms of searching for robust and versatile hemocompatible coatings, as platelet–surface interactions are strongly influenced by nanoscale chemistry and topography. PTYR nanocoatings might thus be more promising than thicker PDA coatings in this respect, and more extensive research is needed to explore their potential in nanoengineered biomaterial applications. In terms of cell adhesion to polycatechol nanocoatings, our study showed that there is a hydrophilicity and roughness threshold above which adhesion and spreading of cells are suppressed rather than enhanced. However, such thresholds most probably vary for different cell types, which must be taken into consideration when designing cell-attracting/repelling surfaces. These findings emphasize the importance of precisely controlled nanoscale surface features in the development of next-generation bioactive coatings for medical implants and blood-contacting devices.

## Experimental

### Materials and methods

#### Stainless steel samples

Stainless steel 316L discs (14 mm in diameter, 0.8 mm in thickness) were laser-cut by STOMILEX (Poland) and tumbler-polished (MARBAD, Poland). Briefly, the discs were placed in a rotating tumbler together with 6 mm ceramic fittings and non-ionic detergent for 18 h. After the tumbling, the discs were rinsed and air-dried.

#### Coating of SS 316 L with polycatecholamines (PDA or PTYR)

The tumbler-polished SS 316L discs were pretreated by rinsing their surfaces with acetone (Chempur, Poland) and a “piranha solution” containing sulfuric acid (Chempur, Poland) and 30% H_2_O_2_ (Sigma-Aldrich, Germany) at a volume ratio of 1:1. The discs were then rinsed in deionized water, followed by immersion in the corresponding coating solution as described (according to patent no P.429942, K. Kopeć, A. Kalinowska, T. Ciach). To create a polydopamine (PDA) coating, samples were immersed into the solution containing 2.0 mg/mL dopamine hydrochloride (Alfa Aesar, Germany) and sodium periodate (Sigma-Aldrich, Germany) at a molar ratio of 1:2 relative to dopamine in 50 mM acetate buffer, at pH adjusted to 5.5. The reaction was conducted for 10 min, 30 min, 1 h, or 4 h. To create a polytyrosine (PTYR) coating, samples were immersed into the solution containing 0.8 mg/mL tyrosine (Carl Roth GmbH, Germany), with 0.6 mM FeCl_2_ (Sigma-Aldrich, Germany), and 30% (v/v) H_2_O_2_ solution (Sigma-Aldrich, Germany) so that the molar ratio of H_2_O_2_/FeCl_2_ was 25:1. The pH of this solution was adjusted to 4.0, and the reaction was conducted for 30 min, 1 h, 4 h, or 24 h. All coating processes were carried out on a magnetic stirrer (300 rpm) at 25.0 °C. Reaction times were preselected in preliminary studies and yielded visually homogenous coatings with short-term stability. After the coating process, discs were washed extensively in deionized water.

#### Wettability of the coatings

Contact angles (CAs) were measured using the sessile drop method (DSA100 goniometer, Kruss GmbH, Germany). A drop of distilled water (5 µL) was placed on a clean and dry surface of a sample. CAs were measured after 60 s with the Kruss DCA 100 software. The measurement was performed at three randomly selected spots on the analyzed material, and each material was prepared in triplicate (*n* = 9 measurements).

The materials obtained with extreme coating times underwent for further tests, that is, PDA 10 min, PDA 24 h, PTYR 30 min, PTYR 24 h.

#### Thickness of the coatings

Microscopic imaging was performed using a Hitachi SU8230 ultrahigh-resolution field-emission scanning electron microscope (Hitachi High-Technologies Corporation, Japan) at an accelerating voltage of 5.0 kV. To measure the thickness of PDA and PTYR coatings, the coated materials were scratched with a scalpel to create a cavity. For imaging, the sample was positioned vertically, and the microscope stage tilt angle was adjusted to visualize the protruding coating layer or the back wall of the cavity. Magnifications ranged from 5000× to 40000×.

#### Roughness analysis

Surface roughness was measured with a DektakXT stylus profilometer from Bruker with the NLite + package. A tip with a radius of 5 µm was used, and the measured length was 1.8 mm. Five locations on each sample were evaluated. The results were analyzed using the vision64 software from Bruker.

#### Sterilization effectiveness of the materials

The sterilization effectiveness of the two different methods on SS-PDA and SS-PTYR materials was evaluated. Two methods of wet sterilization were applied, namely, (a) soaking in a 70% ethanol solution for 30 min, and (b) soaking in a solution containing 100 U/mL penicillin, 100 µg/mL streptomycin, and 0.25 µg/mL amphotericin for 30 min. The exact procedure and the results are described in Supporting Information File 1. For the following biological tests, soaking in 70% ethanol was applied for sterilization.

#### Coating resistance in physiological-like conditions

All SS-PDA and SS-PTYR materials were dipped in PBS in a 24-well plate at 37 °C. Well plates were placed on a microplate shaker (200 rpm) for 1–28 d. The coatings’ surfaces were sputter-coated with a 10 nm layer of AuPd and visualized with an ultrahigh-resolution field-emission scanning electron microscope Hitachi SU8230 (Hitachi High-Technologies Corporation, Japan, magnification 5 000×). The imaging was done before washing and then after 1, 7, 14, and 28 d to evaluate the coating detachment or any morphological changes.

#### Primary amine content evaluation

Evaluation of primary amine (–NH2) content in the coatings was performed using methyl orange assay (adapted from [53]) to link the platelets response to the materials with the chemical composition of the coatings. Briefly, all tested materials were immersed in 0.05% methyl orange (Sigma-Aldrich, Germany) solution in 0.1 M NaH_2_PO_4_ (Chempur, Poland) for 30 min, at RT, on an orbital shaker (200 rpm). Then, the materials were rinsed with 0.1 M NaH_2_PO_4_ and incubated on the orbital shaker at 300 rpm in 300 µL of 0.1 M K_2_CO_3_ (Chempur, Poland) for 10 min, at RT. The absorbance of the resorbed methyl orange solutions was directly proportional to the primary amine group content. Each solution was measured twice at 464 nm. The test was repeated five times. Each material variant was used in triplicate.

To further evaluate the chemical structure of the coatings, FTIR-ATR spectra were obtained and analyzed. Methodology and results are described in Supporting Information File 1.

#### Coating cytotoxicity towards L929 cells

The MTT assay was applied to evaluate the cytotoxicity of obtained coatings. MTT salt is cleaved by mitochondrial dehydrogenase in metabolically active cells and is reduced to an insoluble, purple formazan crystal. Before the test, all materials were sterilized in 70% ethanol for 30 min, rinsed thoroughly with PBS, and air-dried. Then extracts from the materials were prepared according to ISO 10993-11:1993(E). Briefly, sterilized materials were dipped in 1.5 mL of DMEM with 10% FBS and 1% pen-strep, without Phenol Red. The medium amount corresponds with the sample area, according to ISO 10993-11:1993 [54]. Extractions took place after 24, 72, and 168 h (7 d) in 37 °C, 5% CO_2_ with shaking. Each extract was obtained in triplicate.

Mouse fibroblast L929 cells (Sigma-Aldrich, Germany) were expanded according to the manufacturer’s instructions and seeded in a 96-well plate at a density of 105 cells/mL, 100 µL per well. Wells for positive control (DMEM with 0.1% Triton X-100, Gibco, ThermoFisher, USA) and negative control (supplemented DMEM, Gibco, ThermoFisher, USA) were also prepared (PC and NC, respectively). After 24 h of incubation at 37 °C, 5% CO_2_, the medium was discarded from each well, and 100 µL of filtered extracts or controls were added to the wells. After 24 h of incubation at 37 °C, 5% CO_2_ the extracts/controls were discarded, and 50 µL of 1 mg/mL MTT (Sigma-Aldrich, Germany) solution was added to each well. After 4 h of incubation at 37 °C, 5% CO_2_, MTT solution was discarded from wells. The resulting purple formazan crystals were dissolved in each well with 100 µL DMSO. The absorbance of the final purple solutions was read at 570 nm, with reference at 650 nm. As a blank, wells containing L929 cells with added 50 µL of 1 mg/mL MTT and incubated without extracts were used. Each extract was prepared in triplicate and six samples from each extract were measured. Results were expressed as mean % of NC absorbance.

#### Human fibroblast adhesion

Commercially available normal human dermal fibroblasts (PromoCell, Heidelberg, Germany) were cultured in Dulbecco’s modified Eagle’s medium (DMEM, PromoCell, Heidelberg, Germany) supplemented with 10% (v/v) foetal calf serum (FCS) (Sigma-Aldrich, Munich, Germany) and 1% (v/v) penicillin streptomycin (Sigma-Aldrich, Munich, Germany) at 37 °C and a controlled atmosphere of 5% CO_2_ and 95% relative humidity.

Samples were sterilized by immersion in 70% EtOH for 30 min, followed by washing in sterile PBS three times (Gibco, ThermoFisher, USA) before the cell attachment study. Sterilized samples were placed in 24 well plates followed by seeding of 5 × 10^4^ cells per sample and culturing for 1 or 7 d.

At the respective time points, samples were fixed with 4% buffered paraformaldehyde (Roth GmbH, Germany) for 15 min and permeabilized with 0.2% Triton X-100 (Sigma-Aldrich, Germany) for 5 min. Nuclei were stained using Hoechst 33342 at 5 μg/mL final concentration, and F-actin filaments were stained with Alexa 488-phalloidin (Invitrogen, Thermo Fisher, USA). Samples were visualized using a fluorescence microscope Zeiss Axio Observer Z1 (Zeiss, Germany) at 10× magnification. To perform the immunostaining of the N-cadherin adhesion molecule, fibroblasts were seeded on the surface of the materials as described above and cultured for 1 or 7 d. Afterward, samples were fixed with 4% buffered paraformaldehyde for 15 min and permeabilized with 0.2% Triton X-100 for 5 min. All samples were washed with PBS at least three times after the fixation and permeabilization process. A 1% solution of bovine serum albumin was applied to block the samples for 1 h. Primary antibody N-cadherin (13A9) (Santa Cruz Biotechnology, Germany) with 1:200 dilution was applied overnight. Following the washing process, Alexa Fluor 647-conjugated goat anti-rabbit secondary antibody (dilution 1:250, Invitrogen, ThermoFisher, USA) was applied for 1 h. Nuclei and F-actin staining were performed as described above. Samples were visualized using a fluorescence microscope Zeiss Axio Observer Z1 equipped with an AxioCam ICc 1 camera (Zeiss, Germany) at 200× magnification. The surface occupied by the cells and the number of nuclei were determined for each of the six fields of view (FOVs) by ImageJ software [55]. For each sample, six independent FOVs were analyzed. A single FOV corresponded to an area of 184500 µm^2^, yielding a total analyzed area of approximately 1.11 mm^2^.

#### Human platelet adhesion and activation

Blood samples from three healthy volunteers were drawn using Sarstedt S-Monovette coagulation sodium citrate tubes (MSG, Germany). The Ethics Committee of the Faculty of Medicine at the University of Erlangen‐Nürnberg (case no. 21-331-B) approved the use of human material. All subjects enrolled in this research have given informed consent according to the ethical guidelines. Samples were centrifuged at 150*g* for 14 min with 60 s acceleration and 4 min slowdown time. The supernatant was collected and centrifuged again at 150 g for 12 min using the same settings. Platelet-rich plasma (PRP) was transferred to separated tubes. To prevent the loss of platelet function [7], materials were placed into 24-well plates and 200 µL of PRP were added to each sample immediately after PRP separation. Samples were incubated for 90 min at 37 °C and 5% CO_2_. Following the incubation process, samples were washed several times with PBS to remove the unattached platelets and fixed 4% buffered paraformaldehyde. Platelet visualization was performed using an ultrahigh-resolution field-emission scanning electron microscope Hitachi SU8230 (Hitachi High-Technologies Corporation, Japan) at 4500× magnification. Before the visualization, materials were sputter-coated with a 10 nm layer of AuPd. Platelets were then counted per FOV. The activation levels were determined as mild/no activation (round platelet, max. 1–2 pseudopodia) and strong activation (many pseudopodia, “hedgehog-like”). For each sample, eight FOVs were acquired. A single FOV corresponded to an area of 3162 µm^2^, resulting in a total analyzed surface area of 25296 µm^2^ per sample.

#### Statistical analysis

All calculations and graphs were made using OriginPro 9. Before correlation coefficient calculations, all datasets were assessed for normality with Shapiro–Wilk tests, and all *p* values were greater than 0.05; thus, the normality of the data was assumed. The levels of correlation were indicated with Pearson’s correlation coefficient (*r*).

## Supporting Information

File 1 Supplementary methods and results.

## Data Availability

Data generated and analyzed during this study is available from the corresponding author upon reasonable request.
