# Supplementary material for: Polycatecholamine nanocoatings on stainless steel: the effect on attachment of human fibroblasts and platelets
Source: Beilstein J Nanotechnol. 2026 Feb 20;17:365–80. doi: 10.3762/bjnano.17.25 (PMC12927489; doi:10.3762/bjnano.17.25)
Supplement: File 1 — Supplementary methods and results. [file Beilstein_J_Nanotechnol-17-365-s001.pdf]

BEILSTEIN JOURNAL OF NANOTECHNOLOGY

# Supporting Information

for

# Polycatecholamine nanocoatings on stainless steel: the effect on attachment of human fibroblasts and platelets

Paulina Trzaskowska, Ewa Rybak, Maciej Trzaskowski, Kamil Kopeć, Jakub Krzemiński, Rafał Podgórski, Hatice Genc, Mehtap Civelek and Iwona Cicha

*Beilstein J. Nanotechnol.* **2026,** *17,* 365–380. doi:10.3762/bjnano.17.25

# Supplementary methods and results

License and Terms: This is a supporting information file under the terms of the Creative Commons Attribution License (https://creativecommons.org/licenses/by/4.0). Please note that the reuse, redistribution and reproduction in particular requires that the author(s) and source are credited and that individual graphics may be subject to special legal provisions.
The license is subject to the *Beilstein Journal of Nanotechnology* terms and conditions: (https://www.beilstein-journals.org/bjnano/terms)

## Supplementary methods

### *Coating sterilization methods*

The sterilisation effectiveness of SS-PDA and SS-PTY materials was evaluated. Two methods of wet sterilisation were applied: M1 – soaking in a 70% ethanol solution for 30 min, and M2 – soaking in a 100 U/mL penicillin – 100 µg/mL streptomycin – 0.25 µg/mL amphotericin solution for 30 min. Then the samples sterility was tested by direct inoculation using two liquid media: liquid thioglycolate medium – PB1, casein and soy hydrolyzate medium – PB2, and two solid media: agar with casein and soy hydrolysate – PM2, Sabouraud agar with benzylpenicillin and tetracycline – PM3 (all media was purchased from BTL Sp. z o.o., Poland), recommended for this purpose by the European Pharmacopoeia (Ph. Eur., European Pharmacopoeia 9 issued on 15 July 2016, valid from 1 January 2017). 4 mL of PB1 and PB2 medium were added to the wells of 12-well plates, and then one sample of the tested material was added to each well. Three samples from each variant of the tested material were used for each type of medium ($n$ = 3). The plates were shaken at 100 rpm for 60 minutes at room temperature. After that, 100 µL from each well with PB1 medium was inoculated into a Petri dish with PM2 medium, and 100 µL from each well with PB2 medium was inoculated into a Petri dish with PM3 medium. Wells containing 4 mL of PB1 and PB2 media were used as negative controls. As a positive control for the PB1 medium, wells containing 4 mL of this media were used, to which 100 µL of Escherichia coli suspension with a cell density corresponding to a value of 0.5 on the McFarland scale was added. As a positive control for the PB2 medium, wells containing 4 mL of this media were used, to which 100 µL of a swab of microorganisms from human skin into the PB2 medium was added. 12-well plates with liquid media and Petri dishes with solid media were incubated for 14 days at temperatures 37 °C for PB1 and PM2 and 22 °C for PB2 and PM3. Sterility was assessed by measuring optical density ($OD_{600}$) using a SPECTROstar Nano spectrophotometer (BMG LABTECH, Germany) for liquid media (after samples removing) and by observing the growth of microorganisms on the surface of solid media.

## Supplementary results

### *FTIR-ATR spectra of obtained coatings*

FTIR-ATR spectra were collected for bare SS and all PDA and PTYR coating variants. The spectrometer was Thermo Scientific Nicolet IS50 with a SmartOrbit high-performance diamond single bounce ATR accessory. OMNIC 9 software was used to analyse and present the spectral data. 32 scans with a resolution of 2 $cm^{-1}$ were taken at each spot.

Absorption bands in obtained spectra are not very intense due to suspected low thickness of the coatings. According to previous work [1] the thickness of SS-PDA 4 h is approximately 108 nm, SS-PDA 10 m is no thicker than 50 nm, and the thickness of SS-PTYR variants does not exceed 40 nm. Please note that due to low intensity of peaks quantitative analysis was not possible with FTIR. Nevertheless, it was possible to identify peaks that could be assessed to specific bonds in polycatechols (Fig. S1 a)): O–H stretching at 3750 $cm^{-1}$ [2], C-H stretching at 3000–2800 $cm^{-1}$ as well as C=O, N–H and C=C at 1770–1590 $cm^{-1}$ indicating the presence of aromatic amino groups in polydopamine [3]. Thanks to application of advanced ATR mode – an enhancement tool available in OMNIC software, it was possible to subtract bare SS spectrum from the coatings' spectra. As a result, more characteristic signals could be isolated (Fig. S1 b)).: stretching vibration related to N–H and broad O–H peaks at 3300–3000 $cm^{-1}$ [4], peak at 2174 $cm^{-1}$ and double peaks at 1597 and 1500 $cm^{-1}$ attributed respectively to the N–H bonding and stretching vibration of C=C in the aromatic ring [5], and the signal at 1290 $cm^{-1}$ related to the bonding vibration of C–O–H in the catechol moieties [6]. Peak at around 1006 $cm^{-1}$ corresponds to C–O stretching vibration, typical for catechols [4]. Signals in 750–850 $cm^{-1}$ region in SS-PDA 4 h (red) and SS-PTYR 24 h (purple) are less intense than for other variants, which indicates greater decline in aromatic hydrogen amounts in these two coatings as a result of catechol

polymerization [7]. Distinctive peak at 2031–1960 $cm^{-1}$ may, according to the literature, correspond to unsaturated bond between carbon atoms in alkynes [8], or else to allene C=C=C or ketenimine C=C=N, which can draw some more light onto polycatechol polymerization mechanism. Overall, since SS-PDA 10 m and SS-PTYR 30 m are predicably the thinnest, their spectra show the lowest signals intensity.

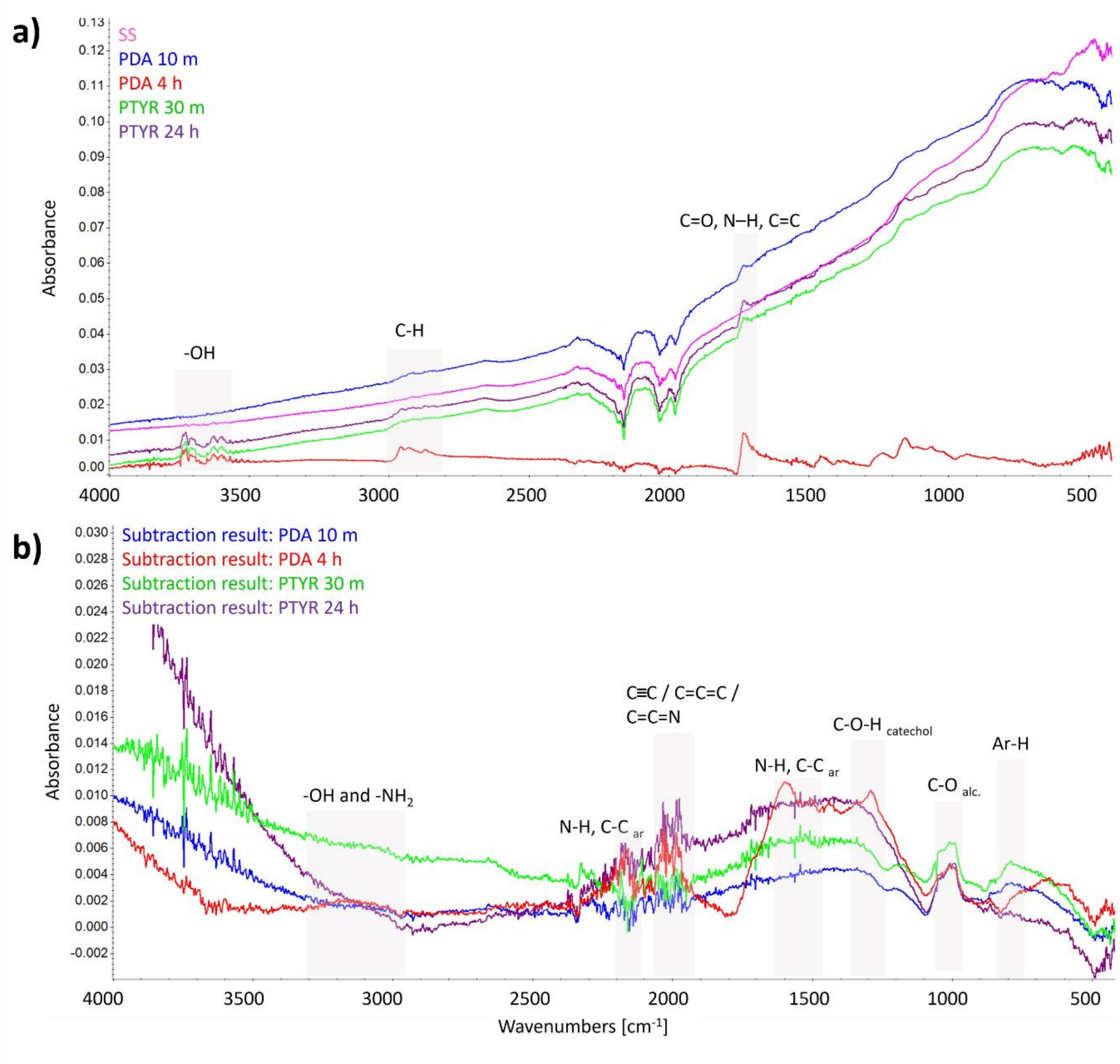


Figure S1. a) FTIR-ATR spectra obtained for SS and variants of SS-PDA and SS-PTYR. b) results of subtraction of bare SS spectrum from SS-PDA and SS-PTYR spectra. Enhanced spectra were used (automatic baseline and advanced ATR mode were applied in OMNIC software).

***Coating sterilization efficacy***

For both tested sterilization methods M1 and M2, no turbidity of both PB1 and PB2 liquid media was observed in all wells with tested samples. This was also confirmed by $OD_{600}$ measurements. The media in the test sample wells had an optical density at the level of the negative controls, as shown in Figure S2. Also, for both tested sterilization methods M1 and M2, no growth of microorganisms was observed on all Petri dishes with PM2 and PM3 media (data not shown). This means that both tested sterilization methods for steel discs with PDA and PTYR coatings are effective.

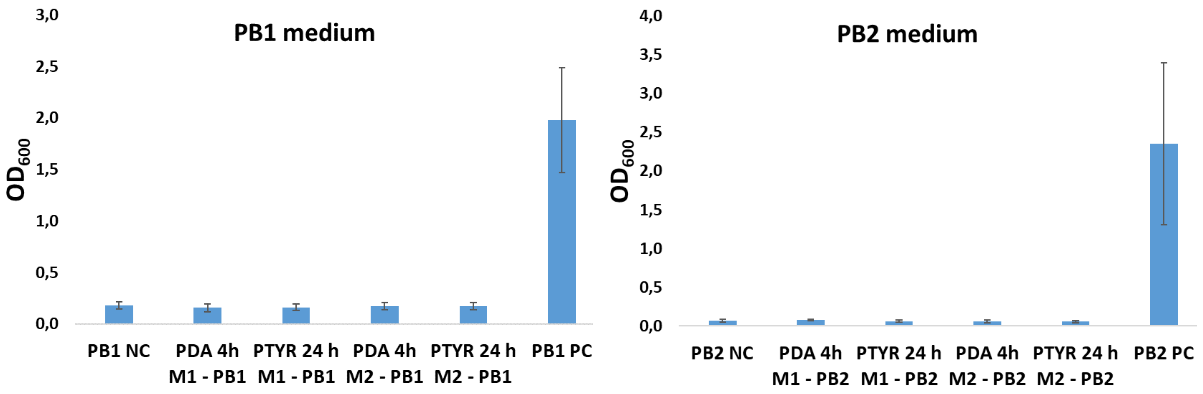


Figure S2. Assessment of the sterility of SS-PDA and SS-PTY materials after sterilization using the M1 and M2 methods - optical density of PB1 and PBA2 media after 14 days of incubation with the tested samples. NC – negative control, PC – positive control.

### *Human platelets adhesion and activation*

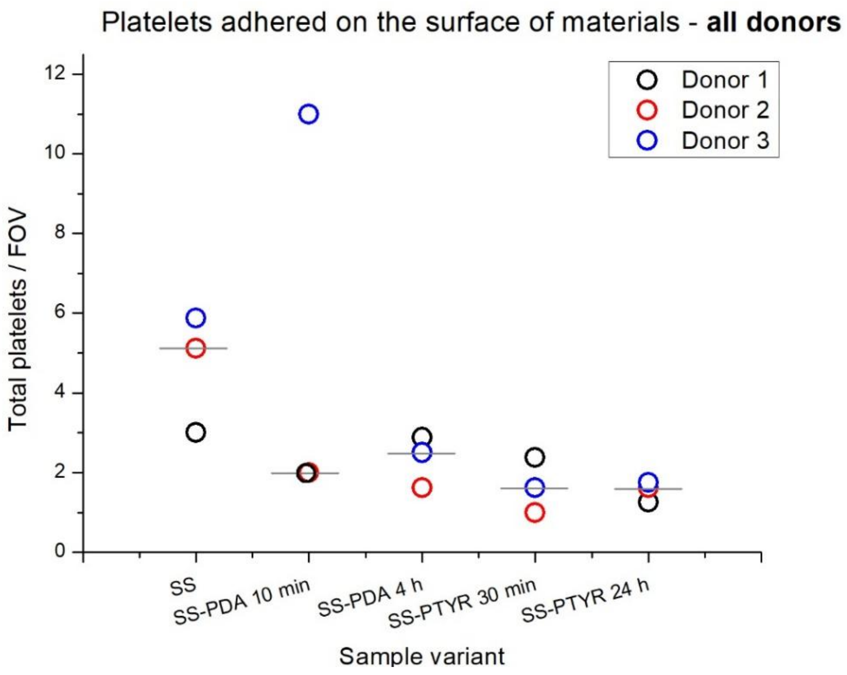


Figure S3. Total numbers of platelets adhered on the surfaces of material variants. The results were obtained using platelet rich plasma from 3 donors. Thin grey lines represent median values of total platelets / FOV (field of view).

*Effect of Rku value on human dermal fibroblasts adhesion*

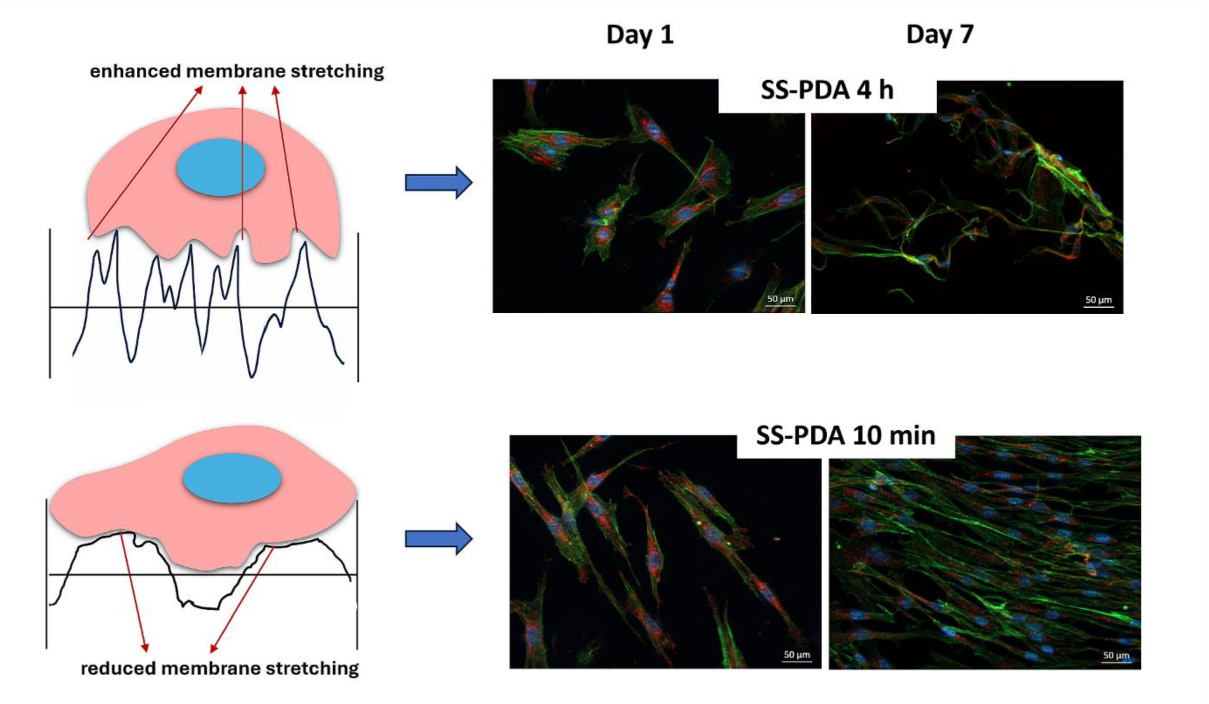


Figure S4. Adhesion of human fibroblasts to material surface in dependence of Rku value. Confocal images of fibroblast adhered to the surface of SS-PDA 4 h or SS-PDA 10 min after 1 day or 7 days of culture are shown: green, F-actin; red, N-cadherin; blue, nuclei. Figure S4 was redrawn from [9].

**References**


1. Kopeć, K.; Ryzko, A.; Major, R.; Plutecka, H.; Więcek, J.; Pikus, G.; Trzciński, J. W.; Kalinowska, A.; Ciach, T. *ACS Omega* **2022,** *7,* 39234–39249. doi:10.1021/acsomega.2c05289
2. Szewczyk, J.; Babacic, V.; Krysztofik, A.; Ivashchenko, O.; Pochylski, M.; Pietrzak, R.; Gapiński, J.; Graczykowski, B.; Bechelany, M.; Coy, E. *ACS Appl. Mater. Interfaces* **2023,** *15,* 36922–36935. doi:10.1021/acsami.3c05236
3. Xavier, J. R. *J. Appl. Electrochem.* **2021,** *51,* 959–975. doi:10.1007/s10800-021-01547-z
4. Mehwish, N.; Xu, M.; Zaeem, M.; Lee, B. H. *Gels* **2022,** *8,* 679. doi:10.3390/gels8100679
5. Thakur, A.; Ranote, S.; Kumar, D.; Bhardwaj, K. K.; Gupta, R.; Chauhan, G. S. *ACS Omega* **2018,** *3,* 7925–7933. doi:10.1021/acsomega.8b01099
6. Yang, Z.; Tu, Q.; Zhu, Y.; Luo, R.; Li, X.; Xie, Y.; Maitz, M. F.; Wang, J.; Huang, N. *Adv. Healthcare Mater.* **2012,** *1,* 548–559. doi:10.1002/adhm.201200073
7. Khosravi, H.; Naderi, R.; Ramezanzadeh, B. *Biomass Convers. Biorefin.* **2022,** *14,* 27557–27573. doi:10.1007/s13399-022-03514-w
8. Mohan Rao, U.; Fofana, I.; Kartheek, R.; Yapi, K. M. L.; Jaya, T. *Lect. Notes. Electr. Eng.* **2020,** *598,* 615–624. doi:10.1007/978-3-030-31676-1_58
9. Bourkoula, A.; Constantoudis, V.; Gogolides, E.; Tserepi, A.; Giakoumakis, G.; Palles, D.; Goustouridis, D.; Tsoukalas, D. *J. Phys. D: Appl. Phys.* **2016,** *49,* No. 304002. doi:10.1088/0022-3727/49/30/304002
